# Leveraging CAR macrophages targeting c-Met for precision immunotherapy in pancreatic cancer: insights from single-cell multi-omics

**DOI:** 10.1186/s10020-024-00996-4

**Published:** 2024-11-26

**Authors:** Lingyu Hu, Xiaoguang Wang, Zhengwei Song, Fei Chen, Bin Wu

**Affiliations:** Department of Surgery, The Second Affiliated Hospital of Jiaxing University, No. 1518 North Huancheng Road, Jiaxing, Zhejiang 314000 People’s Republic of China

**Keywords:** Pancreatic cancer, CAR macrophages, c-Met, Single-cell multi-omics, Angiogenesis, Cancer stem cells

## Abstract

**Background:**

Pancreatic cancer is known for its poor prognosis and resistance to conventional therapies, largely due to the presence of cancer stem cells (CSCs) and aggressive angiogenesis. Effectively targeting these CSCs and associated angiogenic pathways is crucial for effective treatment. This study leverages single-cell multi-omics to explore a novel therapeutic approach involving Chimeric Antigen Receptor (CAR) macrophages engineered to target the c-Met protein on pancreatic CSCs.

**Methods:**

We employed single-cell RNA sequencing to analyze pancreatic cancer tissue, identifying c-Met as a key marker of CSCs. CAR macrophages were engineered using a lentiviral system to express a c-Met-specific receptor. The phagocytic efficiency of these CAR macrophages against pancreatic CSCs was assessed in vitro, along with their ability to inhibit angiogenesis. The in vivo efficacy of CAR macrophages was evaluated in a mouse model of pancreatic cancer.

**Results:**

CAR macrophages demonstrated high specificity for c-Met + CSCs, significantly enhancing phagocytosis and reducing the secretion of angiogenic factors such as VEGFA, FGF2, and ANGPT. In vivo, these macrophages significantly suppressed tumor growth and angiogenesis, prolonging survival in pancreatic cancer-bearing mice.

**Conclusion:**

CAR macrophages targeting c-Met represent a promising therapeutic strategy for pancreatic cancer, offering targeted elimination of CSCs and disruption of tumor angiogenesis. This study highlights the potential of single-cell multi-omics in guiding the development of precision immunotherapies.

## Introduction

Pancreatic cancer, a highly malignant tumor, poses a significant clinical challenge due to its aggressive nature and poor prognosis (Tolliver et al. 2018; Omari et al. 2019). Globally, the incidence and mortality rates of pancreatic cancer are on the rise, making it a major threat to human health (Klein 2021; Sattar et al. 2021; Hu et al. 2021). Early diagnosis is particularly challenging as initial stages often lack clear symptoms, leading to late-stage diagnosis in most patients (Wood et al. 2022; Gao et al. 2023; Kartal et al. 2022). Moreover, the efficacy of conventional treatments, including surgery, chemotherapy, and radiotherapy, is limited due to the tumor’s unique microenvironment and complex molecular mechanisms, rendering these approaches often ineffective (Beutel and Halbrook 2023; Chen et al. 2022; Poh and Ernst 2021). Thus, exploring new therapeutic strategies is imperative.

Although surgical resection is considered one of the most effective treatment methods, most patients miss the optimal timing for surgery at diagnosis (CReST Collaborative Group 2022). Chemotherapy and radiotherapy, while extending survival to some extent, are limited by the high heterogeneity of pancreatic cancer cells and their resistance to treatment (Tang et al. 2022; Li et al. 2023; Wang et al. 2022). Additionally, targeted therapy faces challenges due to the complex and variable molecular characteristics of pancreatic cancer, underscoring the need for novel therapeutic approaches, particularly those targeting the unique features of pancreatic cancer, to improve patient survival rates (Newhook et al. 2023; DiPeri et al. 2022).

The advancement of biotechnology has positioned single-cell multi-omics as a powerful tool for uncovering tumor heterogeneity and the complexity of the microenvironment. By analyzing gene expression, protein levels, and epigenetic states of individual cells, researchers can gain deep insights into the biology of tumors, leading to the identification of new therapeutic targets (Baysoy et al. 2023; Liu et al. 2023; Hsieh et al. 2022). In pancreatic cancer research, the application of single-cell multi-omics facilitates the identification of different cancer cell subtypes, the composition of immune cells in the tumor microenvironment, and their interactions, offering the potential for the development of more targeted therapeutic strategies (Fan et al. 2022; Zhang et al. 2020a, b).

Recently, Chimeric Antigen Receptor (CAR) technology has made significant strides in cancer treatment, especially with the success of CAR-T cell therapy in certain hematological malignancies (Sterner and Sterner 2021; Zhang et al. 2022; Pan et al. 2022; Zhang et al. 2020,b). In contrast, CAR macrophage (CAR-M) therapy, an emerging strategy, shows immense potential for treating solid tumors, including pancreatic cancer (Mishra and Malonia 2023; Schepisi et al. 2023). CAR-M therapy, by engineering macrophages to specifically recognize and phagocytize cancer cells while modulating the tumor microenvironment to promote anti-tumor immune responses, presents a new hope in combating pancreatic cancer (Maalej et al. 2023; Chen et al. 2021; Sloas et al. 2021). Particularly, the development of CAR macrophages targeting specific molecules on the surface of pancreatic cancer cells, such as c-Met, offers promising prospects.

This study aims to delve into the molecular mechanisms of pancreatic cancer and develop CAR macrophage therapy targeting pancreatic CSCs, utilizing single-cell multi-omics technology. We focus on the overexpressed molecular marker c-Met in pancreatic cancer cells, developing specific CAR-M cells to inhibit angiogenic mimicry formation and tumor growth. Through in vitro and in vivo experiments validating the efficacy of this therapy, our study not only provides new strategies for treating pancreatic cancer but also serves as a reference for treating other solid tumors. We believe the development and application of CAR macrophage therapy will bring new treatment hopes to pancreatic cancer patients, significantly improving their survival rates and quality of life.

## Materials and methods

### Transcriptomic data acquisition and preprocessing

Initially, pancreatic cancer-related transcriptomic sequencing datasets, including 69 pancreatic cancer tissue samples and 61 adjacent non-tumor samples, were downloaded from the Gene Expression Omnibus (GEO, https://www.ncbi.nlm.nih.gov/geo/) database (dataset GSE62452). The datasets underwent standardization using the limma package (version 3.44.3) in R software for background correction and quantile normalization, ensuring the data quality was suitable for subsequent analyses.

### Differential gene expression analysis

Differential expression analysis was conducted using the limma package, setting a threshold for statistical significance (adjusted *P*-value < 0.05 and |log2(fold change)| > 1) to identify genes with significant expression level changes in pancreatic cancer tissues compared to normal tissues.

### Functional and pathway enrichment analysis of differentially expressed genes

To interpret the biological significance of the differentially expressed genes (DEGs), gene ontology (GO) and Kyoto Encyclopedia of Genes and Genomes (KEGG) pathway enrichment analyses were performed using the ‘clusterProfiler’ package in R. Enrichment significance threshold was set at *p*-value < 0.05 for both GO terms and KEGG pathways to elucidate the functions and related biological processes of these genes in pancreatic cancer.

### GSEA enrichment analysis

The GSE62452 dataset, which contains gene expression data from pancreatic cancer samples, was utilized for conducting a GSEA enrichment analysis. The samples were classified into high and low c-MET expression groups, and all genes were ranked by differential expression to create a list from significantly upregulated to downregulated genes. Enrichment scores (ES) for gene sets from the “Hallmark gene sets” in the MSigDB database were calculated based on this ranked list: the ES increased when genes in the set appeared in the ranked list and decreased otherwise, with the maximal enrichment point marking the peak or trough of the curve.

### Single-cell RNA sequencing data acquisition and preprocessing

Single-cell RNA sequencing datasets related to pancreatic ductal adenocarcinoma, including 6 tumor samples and 6 adjacent non-tumor samples, were downloaded from the GEO database (dataset GSE212966). Samples GSM6567167, GSM6567169, and GSM6567170 were excluded from the analysis due to missing gene expression information. The Cell Ranger software (version 3.0.2, 10x Genomics) was utilized for quality control and generation of gene expression matrices from raw sequencing files.

### Data dimensionality reduction, clustering, and cell population annotation

The Seurat package (version 3.2.1, https://satijalab.org/seurat/) was employed for quality control and normalization of the raw single-cell RNA sequencing data. Cells with low gene expression and abnormal gene count detection were removed to ensure data quality and consistency. Principal component analysis (PCA) was applied to reduce data dimensionality and highlight major variations between cells. Key principal components were selected for subsequent clustering and visualization analyses. A graph-based clustering approach was used to identify natural cell clusters. The resolution parameter was adjusted to determine the optimal number of cell clusters, ensuring the clustering results had biological significance. Each cell cluster was annotated based on known cell type marker genes to identify different cell types and states, referencing public cell type annotation databases to enhance the accuracy and completeness of annotations.

### Analysis of cell population proportion changes

An analysis of cell population proportion changes in normal and cancerous tissues was conducted using R software (version 4.0.3, https://cran.r-project.org/). By comparing the proportions of immune cells between normal and cancerous tissues, insights into the immunological microenvironment changes in gallbladder cancer were obtained.

### Identification of malignant ductal cell marker gene sets in pancreatic cancer

The FindAllMarkers function in Seurat was used to identify specific marker genes that distinguished malignant ductal cells in pancreatic cancer from normal ductal cell populations. Pancreatic cancer-related genes were searched for in GeneCards (https://genecards.org/) and genes with a Relevance score > 100 were intersected with previously analyzed differentially expressed genes to identify core genes.

### Prognostic evaluation based on c-Met expression

A survival analysis was conducted using clinical data and c-Met expression levels from pancreatic cancer patients available in the Kaplan-Meier Plotter database (https://kmplot.com/analysis/). The survival rate differences between patient groups with high and low c-Met expression were assessed using Kaplan-Meier plots, with statistical significance calculated using the log-rank test and the significance level set at a P-value < 0.05.

### Development of c-Met-specific CARs

SFFV_ΔCAR and SFFV_CAR vectors were acquired from Addgene (#113014 and #113019). Initially, the CAR sequence from the SFFV_CAR vector was cloned into the third-generation self-inactivating (SIN) lentiviral backbone (pRRL.cPPT.CBX3.EFS), creating the CBX3.EFS.CAR vector. Subsequently, an internal ribosome entry site (IRES) linked to an enhanced green fluorescent protein (eGFP) reporter sequence was inserted at the SalI site within the pRRL.cPPT.CBX3.EFS.CAR vector. Finally, the anti-CD19 sequence in the SFFV ΔCAR and SFFV CAR vectors was replaced with an anti-c-Met scFv sequence (patent number: CN104159909A) using the linearized lentiviral vector plasmid GV401 (Shanghai Genechem Co. Ltd., China). The designed lentiviral ΔCAR/CAR vector plasmids were verified by DNA sequencing (GATC and SeqLab) (Abdin et al. 2023; Min et al. 2022).

### Virus production

Lentiviral vector particles were produced using HEK293T cells (ATCC) and cultured as per the official guidelines. HEK293T cells were transfected using the calcium phosphate method with 8 µg/mL pcDNA3.1 (gag/pol) (V79020, ThermoFisher), 5 µg/mL pRSV-Rev (#12253, Addgene), 5 µg/mL lentiviral eGFP, ΔCAR, or CAR vector plasmid, and 2 µg/mL vesicular stomatitis virus G protein (VSVg) plasmid pMD2.G (BR037, Fenghui Biotechnology). Cell supernatant was filtered through a 2 μm filter and concentrated overnight at 4 °C, 10,000×g using an Avanti J-26XP centrifuge (Beckman Coulter, Brea, CA, USA). The lentiviral vector titer was determined using a protocol involving serial dilutions (1:100, 1:1000, and 1:10,000) of viral particles in a culture medium supplemented with protamine sulfate (4 µg/mL, P3369) to transduce 1 × 10^5^ adherent SC-1 cells (ml-A1843, MlBio). Seventy-two hours post-transfection, eGFP fluorescence intensity was analyzed using a CytoFLEX S flow cytometer (Beckman Coulter, Brea, CA, USA) to determine viral titer and transduction efficiency (Paasch et al. 2022).

### Inducing macrophage differentiation from pluripotent stem cells

Induced pluripotent stem cells (iPSCs) were obtained from ATCC (ACS-1020) and grouped for infection as follows: the eGFP group (transfected with a control vector expressing eGFP), the ∆CAR group (transfected with a truncated CAR vector), and the CAR group (transfected with a full-length CAR vector). Corresponding concentrated viral fluids were used to infect 1 × 10^6^ iPSCs at a multiplicity of infection (MOI) of 5. After 48 h, cells expressing eGFP were sorted using a FACSAria Fusion flow cytometer (BD Biosciences, Franklin Lakes, NJ, USA) to select successfully infected cells. The sorted cells were cultured in an E8 medium for 48 h before initiating macrophage differentiation. Undifferentiated iPSCs were treated with Versene (15040066, Gibco) for 6 minutes, then transferred to low-attachment plates (3471, Corning). Embryoid bodies (EBs) were formed by overnight incubation at 37 °C, 5% CO2, and 50 rpm in mTeSR medium (85850, STEMCELL Technologies). On day 1 post-EB formation, primitive streak mesoderm precursors were cultured in APEL II medium (05270, STEMCELL Technologies) supplemented with BMP-4 (10 ng/ml) (120-05, PeproTech) and bFGF (5 ng/ml) (100-18B, PeproTech). From days 2–7, the hematopoietic specification was directed using APEL II medium containing BMP-4 (10 ng/ml), bFGF (5 ng/ml), VEGF (50 ng/ml) (100 − 20, PeproTech), and SCF (100 ng/mL) (300-07, PeproTech). On days 8–9, myeloid differentiation was induced by adding bFGF (10 ng/ml), VEGF (50 ng/ml), SCF (50 ng/mL), IGF-1 (10 ng/mL) (100 − 11, PeproTech), IL-3 (25 ng/mL) (200-03, PeproTech), M-CSF (50 ng/mL) (300 − 25, PeproTech), and GM-CSF (50 ng/mL) (300-03, PeproTech) to the APEL II medium. On day 10, 40–50 EBs were placed in 6-well plates pre-coated with Matrigel Matrix (354277, Corning), and the medium was switched to StemPro™-34 SFM (10639011, Gibco) containing bFGF (5 ng/ml), VEGF (50 ng/ml), SCF (50 ng/mL), IGF-1 (10 ng/mL), IL-3 (25 ng/mL), M-CSF (50 ng/mL), and GM-CSF (50 ng/mL) for an additional 10 days. On day 20, suspended cells were collected and reseeded onto new Matrigel plates in StemPro™-34 SFM medium supplemented with bFGF (5 ng/ml), VEGF (50 ng/ml), SCF (50 ng/mL), IGF-1 (10 ng/mL), IL-3 (25 ng/mL), M-CSF (100 ng/mL), and GM-CSF (100 ng/mL) for 2 days, then switched to medium without IL-3 and continued culture until day 27. Differentiation was completed by day 28, yielding iPSC-derived macrophages maintained in RPMI 1640 medium (36750, STEMCELL Technologies) supplemented with M-CSF (100 ng/mL) and GM-CSF (100 ng/mL) (Paasch et al. 2022; Zhang et al. 2020, b).

### Flow cytometry analysis of induced differentiated macrophages

Macrophage surface markers and reporter genes were analyzed using a CytoFLEX S flow cytometer (Beckman Coulter, Brea, California, USA) and antibodies: hCD45-PE-Cy7 (cat. 25–0459), hCD11b-APC (17-0118-41), hCD14-PE (12-0149-41), hCD163-APC (17-1639-41) (eBioscience). Cells were blocked with human FC-Block TruStain FcX (BioLegend) for 5 min at room temperature before staining as per the manufacturer’s instructions. Flow cytometry data were analyzed using FlowJo V.10 (Abdin et al. 2023).

### Evaluating macrophage phagocytosis of cancer cells via flow cytometry

Before adding 1.5 × 10^5^ mCherry + Raji cells (CBP60272, Nanjing Cobioer Biotech), macrophages transfected with the eGFP control vector, or ∆CAR or CAR vectors were stimulated with 500 µg/ml lipopolysaccharide (LPS) (SMB00704, Sigma). After co-culturing effector (E: macrophages) and target (T: Raji cells) cells at a 1:1 ratio, supernatant and cells were collected and passed through a 70 μm filter. Adherent macrophages were washed with PBS, incubated with 500µL Accutase (A1110501, ThermoFisher) at 37 °C for 20 min, then transferred to fluorescence-activated cell sorting tubes. Cells were analyzed using either a CytoFLEX S or an image-based flow cytometer (Flowcyte, Amnis). Macrophages were pre-gated on the eGFP signal, and the percentage of cells within the gate expressing the mCherry signal was analyzed to determine macrophage phagocytic capability (Abdin et al. 2023).

### Evaluation of macrophage phagocytosis using confocal microscopy

In 24-well suspension plates, 2 × 10^5^ macrophages transfected with different CAR vectors were seeded on a 12 mm round coverslip in 500 µL of standard medium containing 100 ng/mL hM-CSF. Three hours prior to adding 2 × 10^5^ mCherry + Raji cells (E: T ratio of 1:1), eGFP-, ∆CAR-, or CAR-macrophages were stimulated with 500 ng/mL LPS (SMB00704, Sigma). After 4 h of co-culture, the supernatant was removed, and the wells containing the coverslips were gently washed twice with 500 µL PBS. The macrophages on the coverslips were then fixed and mounted using ProLong Gold Antifade reagent (Life Technologies). Images were captured with a Leica TCS SP8 inverted microscope (Leica Microsystems) and analyzed using LAS X software. The phagocytic capability was assessed by observing and quantifying the Expression of mCherry within eGFP + macrophages (Abdin et al. 2023).

### ELISA for angiogenic factors

Concentrations of VEGFA (Thermo, KHG0112), FGF2 (Thermo, EB2RB), and ANGPT (Thermo, KHC1641) in the supernatant were measured using commercial ELISA kits. Supernatants from macrophages transfected with different CARs at various time points post-culture were collected, and total protein was extracted. Cell numbers for each experimental group were assessed using the bicinchoninic acid assay. The protein expression levels of VEGFA, FGF2, and ANGPT were normalized and presented as mean ± SD (Lin et al. 2019).

### Tube formation assay

The angiogenic capability of HUVECs (Item No. 7-1074, Chi Scientific) in vitro was tested using a Matrigel tube formation assay. Matrigel (354277, 50 µl/well, Corning) was added to pre-chilled 96-well plates and allowed to polymerize at 37 °C for 2 h. HUVECs were suspended in a mixture of 2% FBS and the supernatant from eGFP, ∆CAR, or CAR macrophage cultures and seeded onto the prepared Matrigel in 96-well plates at a density of 3 × 10^4^ cells per well. After 15 h of culture, cells were stained with FITC-anti-F-actin antibody (F3046, Sigma). Fluorescence microscopy was used to capture images in 3–5 fields of view, and the total length of tube structures was analyzed using the Angiogenesis Analyzer plugin for ImageJ software (Ma et al. 2021).

### Pancreatic transplant tumor model

A 1:1 mixture of Matrigel and 1× PBS was prepared, and 5 × 10^4^ KPC cells (Ximbio, cultured according to the manual) were suspended in it for transplantation into the pancreas of 6–8 week-old female NSG mice (005557, JAX). The cell suspension was injected into the tail region of the pancreas using a Hamilton syringe, with successful injection indicated by the appearance of a blister without intraperitoneal leakage. The abdominal wall was sutured with absorbable Vicryl suture (VR416, Ethicon), and the skin was closed with wound clips (204–1000, CellPoint Scientific). One week post-transplantation, mice were randomly divided and injected with eGFP-, ∆CAR-, or CAR-macrophages suspended in PBS, forming the eGFP-M, ∆CAR-M, and CAR-M groups. The survival status of all mice was tracked from the injection of KPC cells to the end of the study. Survival times were analyzed using the Kaplan-Meier method, and survival curves were plotted and compared using SPSS statistical software. Post-mortem pancreatic tumor tissues were collected for subsequent experiments (Chibaya et al. 2023; Yang et al. 2023).

### Bioluminescence imaging for tracking KPC tumor cells in vivo

KPC tumor cells were transfected with the MSCV-luciferase (luc)-IRES-GFP retroviral vector (MSCV-IRES-GFP, Plasmid #20672; MSCV-IRES-luciferase, Plasmid #18760, Addgene) synthesized by a plasmid company upon consultation. The retrovirus was produced by co-transfecting 293T cells with VSV-G. Post-transfection, GFP + cell populations were purified via FACS sorting using FACSAria (BD Biosciences). Cells were cultured at 37 °C in a 5% CO_2_ humidified incubator in DMEM containing 10% FBS and 100 IU/ml penicillin/streptomycin (P/S) (#36250, STEMCELL Technologies). The KPC cell line was cultured in dishes coated with 100 µg/ml collagen (PureCol, #5005, Advanced Biomatrix).

Bioluminescence imaging (BLI) was utilized to track the Expression of Luciferase-GFP reporter genes in KPC tumor cells post-intravenous injection, enabling analysis of tumor burden across different sites. Mice were injected intraperitoneally with luciferase substrate (5 mg/mouse; LUCK-100, GOLDBIO), and images were captured 10–15 min later using a Xenogen IVIS Spectrum imaging system (PerkinElmer), with an exposure time of 60 s. Quantitative analysis of the luciferase signal within target organ areas was conducted using Living Image software version 7.4.3 (Caliper Life Sciences) (Chibaya et al. 2023).

### Immunofluorescence assay

For tissue immunofluorescence, samples were fixed in 4% PFA, dehydrated in 30% sucrose solution for 24 h, embedded in Tissue-Tek OCT compound, and then cryosectioned into 10 μm thick slices. For cellular immunofluorescence staining, endothelial cells fixed in 4% PFA were permeabilized with 0.5% Triton for 5 min, followed by blocking in 1×PBST containing 5% goat serum for 1 h. Samples were incubated overnight at 4 °C with antibodies against CD31 (ab182981, Abcam), collagen IV (ab236640, Abcam), α-SMA (#19245, CST), and CD11b (379902, Biolegend). After washing 3 times with 1×PBS, secondary antibodies (Donkey anti-mouse 488, A-21202; Donkey anti-rabbit 488, A-21206; Donkey anti-rabbit 555, A-31572; ThermoFisher) were applied for 1-hour incubation at room temperature. Nuclei were stained with DAPI (Beyotime), and samples were mounted with Fluorescence Mounting Medium-G (Southern Biotech). Confocal microscopy (FV3000, Olympus) was used to capture immunofluorescence images, which were analyzed using ImageJ 1.49v software to measure the fluorescence intensity of target proteins, evaluating branching index, pericyte coverage, basement membrane coverage, and infiltration degree. Statistical quantification was performed using SPSS software, with data presented as mean ± SEM. Statistical significance was determined using two-tailed Student’s t-tests, with p-values < 0.05 considered significant (Xu et al. 2021).

### Vascular perfusion and leakage detection experiment

To assess vascular permeability, mice were intraperitoneally injected with 100 µL of 25 mg/mL FITC-conjugated Dextran (53471, Sigma), followed by perfusion with 1% PFA after 30 min to remove circulating polymers. Vascular perfusion was measured by intraperitoneally injecting 100 µL of 1 mg/mL DyLight 488-conjugated Tomato Lectin (DL-1174-1, Vector Laboratories) 30 min before sacrifice. Tumor tissues collected post-treatment were embedded and cryosectioned using Tissue-Tek OCT Compound, and confocal microscopy (FV3000, Olympus) was employed to observe and capture immunofluorescence images. ImageJ 1.49v software was used to measure the fluorescence intensity of target molecules, analyzing the rates of vascular perfusion and leakage, with statistical quantification performed using SPSS software. Data are presented as mean ± SEM, and statistical significance was assessed using two-tailed Student’s t-tests, with p-values < 0.05 deemed significant (Xu et al. 2021).

### Statistical analysis

Data were derived from at least three independent experiments and expressed as mean ± standard deviation (Mean ± SEM). Comparisons between the two groups were conducted using two independent sample t-tests. For comparisons involving three or more groups, one-way ANOVA was employed, followed by Tukey’s HSD post-hoc test for significant differences, for non-normally distributed data or data with unequal variances, Mann-Whitney U tests, or Kruskal-Wallis H tests were used. All statistical analyses were performed using GraphPad Prism 9 (GraphPad Software, Inc.) and R language. The significance level for all tests was set at 0.05, with two-sided p-values < 0.05 considered statistically significant.

## Results

### Identification and functional enrichment analysis of differentially expressed genes unveils key molecular mechanisms in pancreatic cancer

**Fig. 1:**
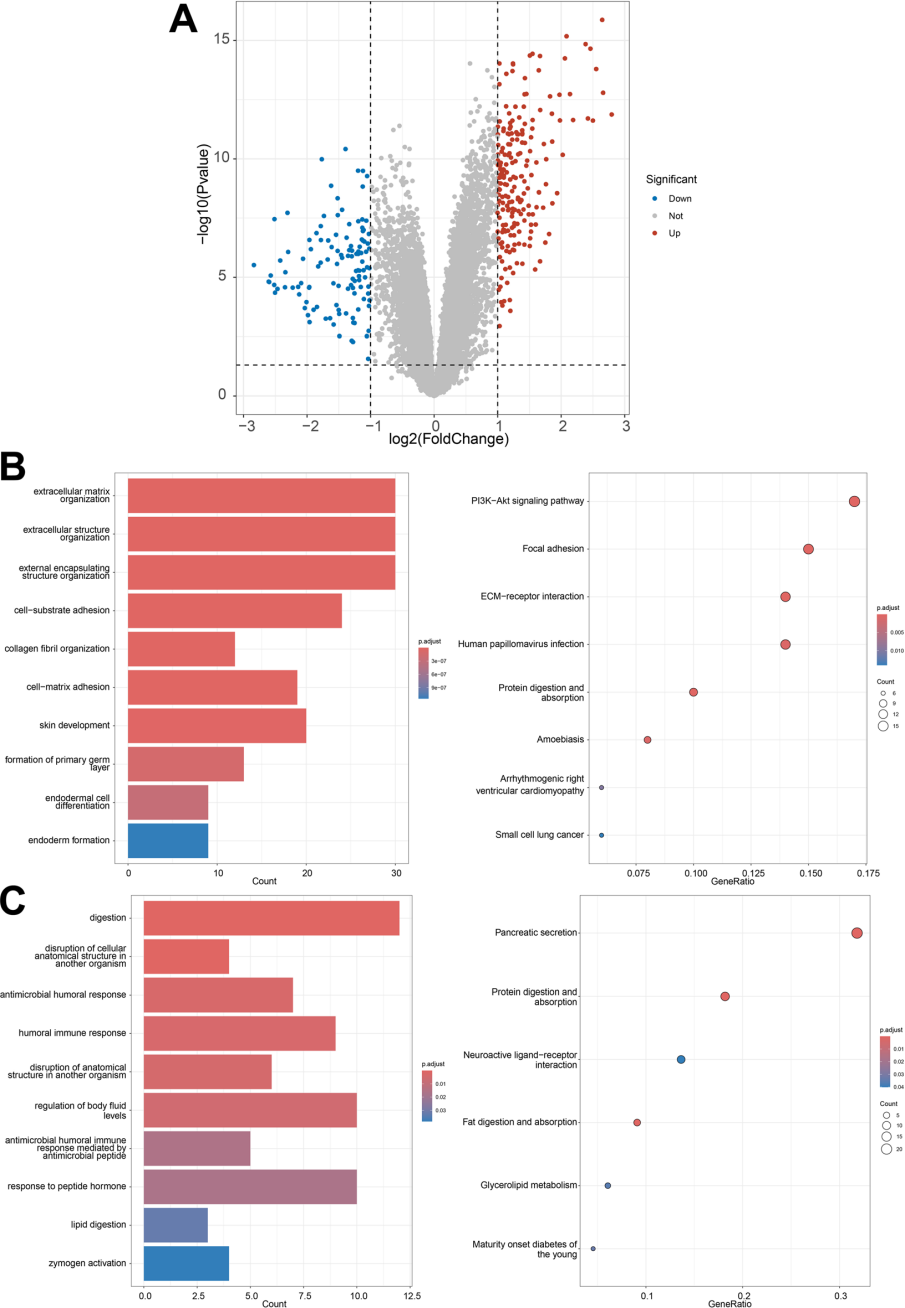
Differential gene expression analysis in pancreatic cancer transcriptome and functional enrichment analysis of key genes. *Note*: (**A**) Identification of 308 differentially expressed genes (DEGs) through transcriptome analysis, including 198 upregulated and 110 downregulated genes. (**B**) Enrichment analysis of upregulated genes in pancreatic cancer. (**C**) Enrichment analysis of downregulated genes in pancreatic cancer

In preprocessing transcriptomic data, the limma package in R software facilitated standardization, yielding high-quality control parameters. Setting specific thresholds identified 308 differentially expressed genes, with 198 genes upregulated and 110 downregulated, as illustrated in Fig. 1A. Enrichment analysis indicated that upregulated genes were predominantly associated with extracellular matrix organization, cell structure, and the PI3K-Akt pathway, while downregulated genes were enriched in digestion and pancreatic secretion pathways (Fig. 1B-C). The activation of these pathways directly correlates with the malignant progression and Angiogenesis of pancreatic cancer, offering new insights into its molecular mechanisms.

### Single-cell analysis reveals immune microenvironment activation and core genes in pancreatic cancer

**Fig. 2:**
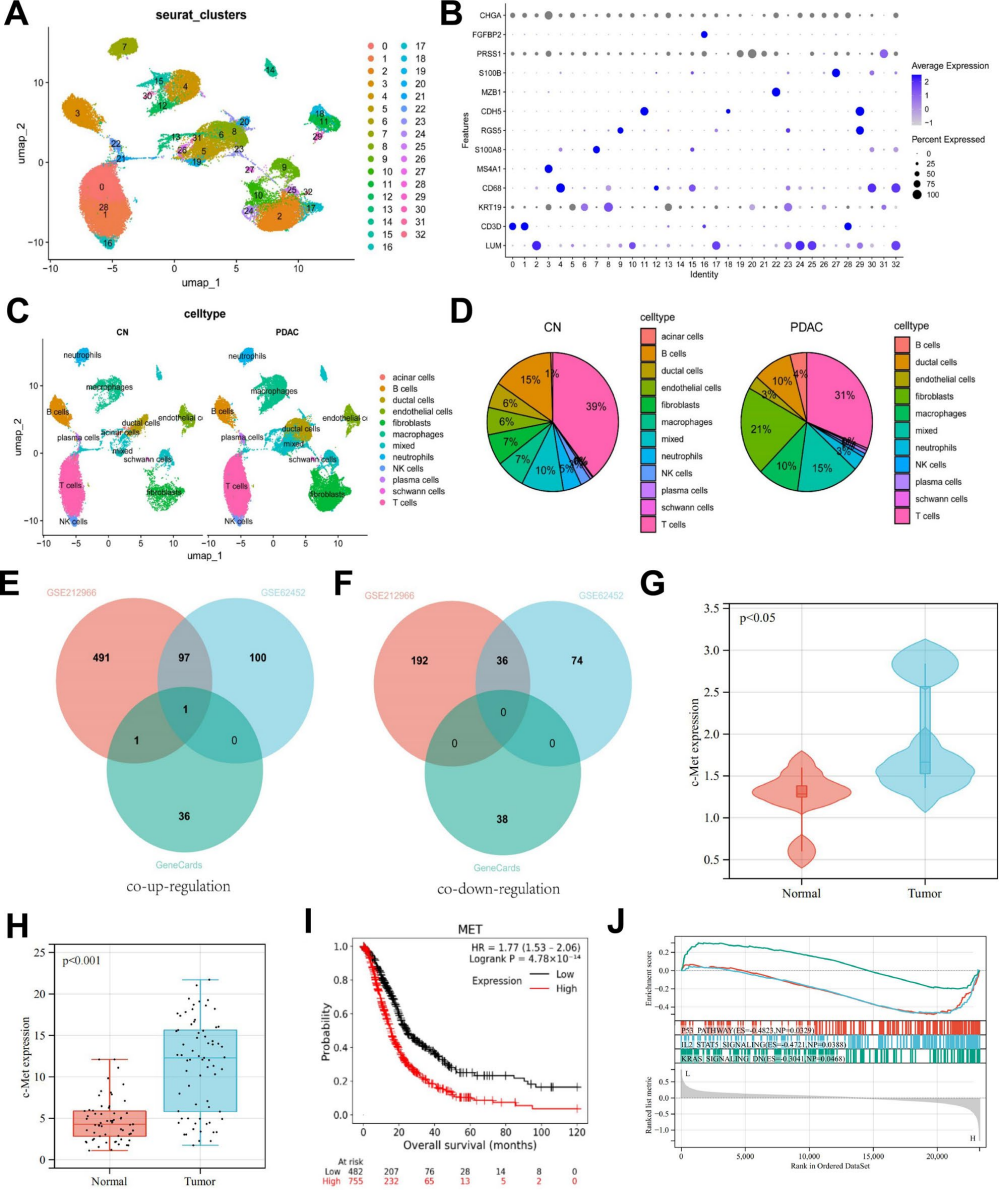
Overview of single-cell RNA sequencing analysis in pancreatic cancer and survival curve analysis. *Note*: (**A**) Clustering results of cells at a resolution parameter of 1.0. (**B**) Annotation of different cell populations based on marker genes, such as LUM and CD3D. (**C**–**D**) Comparison of the proportion of different cell populations in normal and pancreatic cancer tissues, highlighting the differences in cell composition. (**E**–**F**) The Venn diagram shows the intersection of pancreatic cancer differential genes from GSE62452, ductal cell differential genes from GSE212966, and pancreatic cancer-related genes from GeneCards. (**G**) Analysis of c-MET expression levels across different groups based on the single-cell RNA sequencing dataset GSE212966. (**H**) Analysis of c-MET expression levels across different groups based on the transcriptomic dataset GSE62452. (**I**) Kaplan-Meier survival curves comparing overall survival between high and low c-MET expression groups, highlighting the significant association between high c-MET expression and poor prognosis in pancreatic cancer patients. (**J**) GSEA enrichment analysis based on the transcriptomic dataset GSE62452, grouped by high and low c-MET expression

The single-cell RNA sequencing dataset GSE212966, related to pancreatic cancer, was successfully downloaded from the GEO database and processed using Cell Ranger software for quality control and gene expression matrix generation. High-quality cell samples were obtained, and further clustering analysis with the Seurat package identified up to 33 distinct cell populations. Adjusting the resolution parameter revealed that a resolution of 1.0 yielded the most biologically meaningful clustering results (Fig. 2A). The distribution of cell numbers in each cluster varied significantly, highlighting the diversity of the tumor microenvironment. Subsequently, known marker genes annotated distinct cell populations, including fibroblasts (LUM), T cells (CD3D), ductal cells (KRT19), macrophages (CD68), B cells (MS4A1), neutrophils (S100A8), stellate cells (RGS5), endothelial cells (CDH5), plasma cells (MZB1), Schwann cells (S100B), acinar cells (PRSS1), NK cells (FGFBP2), and endocrine cells (CHGA) (Fig. 2B-C).

Further, we compared cell population proportions in normal and pancreatic cancer tissues, finding that macrophages, ductal cells, and fibroblasts increased in cancer tissues, while B and T cells decreased (Fig. 2D). Using the FindAllMarkers function, we identified distinct marker genes for pancreatic cancer ductal cells, revealing that c-MET was the only remaining gene after intersecting with pancreatic cancer-associated genes from GeneCards (Fig. 2E-F). Details of c-MET expression levels are presented in Fig. 2G-H. c-MET, a tyrosine kinase receptor on the cell surface, is implicated in tumor growth, spread, and resistance. Studies indicate c-MET signaling affects macrophage function, promoting tumor support via secretion of factors that enhance tumor proliferation and metastasis (Chen et al. 2019).

To analyze the correlation between c-MET expression and prognosis, we used the Kaplan-Meier Plotter database, showing that pancreatic cancer patients with high c-MET expression (upper quartile) had significantly lower survival rates than those with low expression (lower quartile), with a log-rank test p-value < 0.05. This result identifies high c-MET expression as an independent predictor of poor prognosis (Fig. 2I).

Using transcriptomic data from GSE62452, our single-gene GSEA analysis (Fig. 2J) revealed that in high c-MET expression samples, the p53 pathway activity was suppressed, likely facilitating tumor survival and proliferation, as p53 typically acts as a tumor suppressor (Zhang et al. 2020, b). Simultaneously, the IL2-STAT5 signaling pathway’s reduced activity suggests an immune evasion mechanism by the tumor cells (Lutz et al. 2023). Additionally, low expression of the KRAS signaling downregulation pathway, indicated by the reverse pathway analysis, hints at abnormal activation of the KRAS pathway in these tumor samples (Singhal et al. 2024). These findings collectively suggest that high c-MET expression may promote tumor progression by impacting essential cancer-related pathways.

In conclusion, our single-cell RNA sequencing analysis demonstrated increases in macrophages, ductal cells, and fibroblasts, with reductions in B and T cells in pancreatic cancer tissues. Specifically, high c-MET expression in pancreatic cancer ductal cells was associated with poor prognosis, underscoring its significance in tumor progression.

### Generation and phagocytosis of iPSC-derived macrophages targeting c-Met + tumor cells via CAR technology

**Fig. 3:**
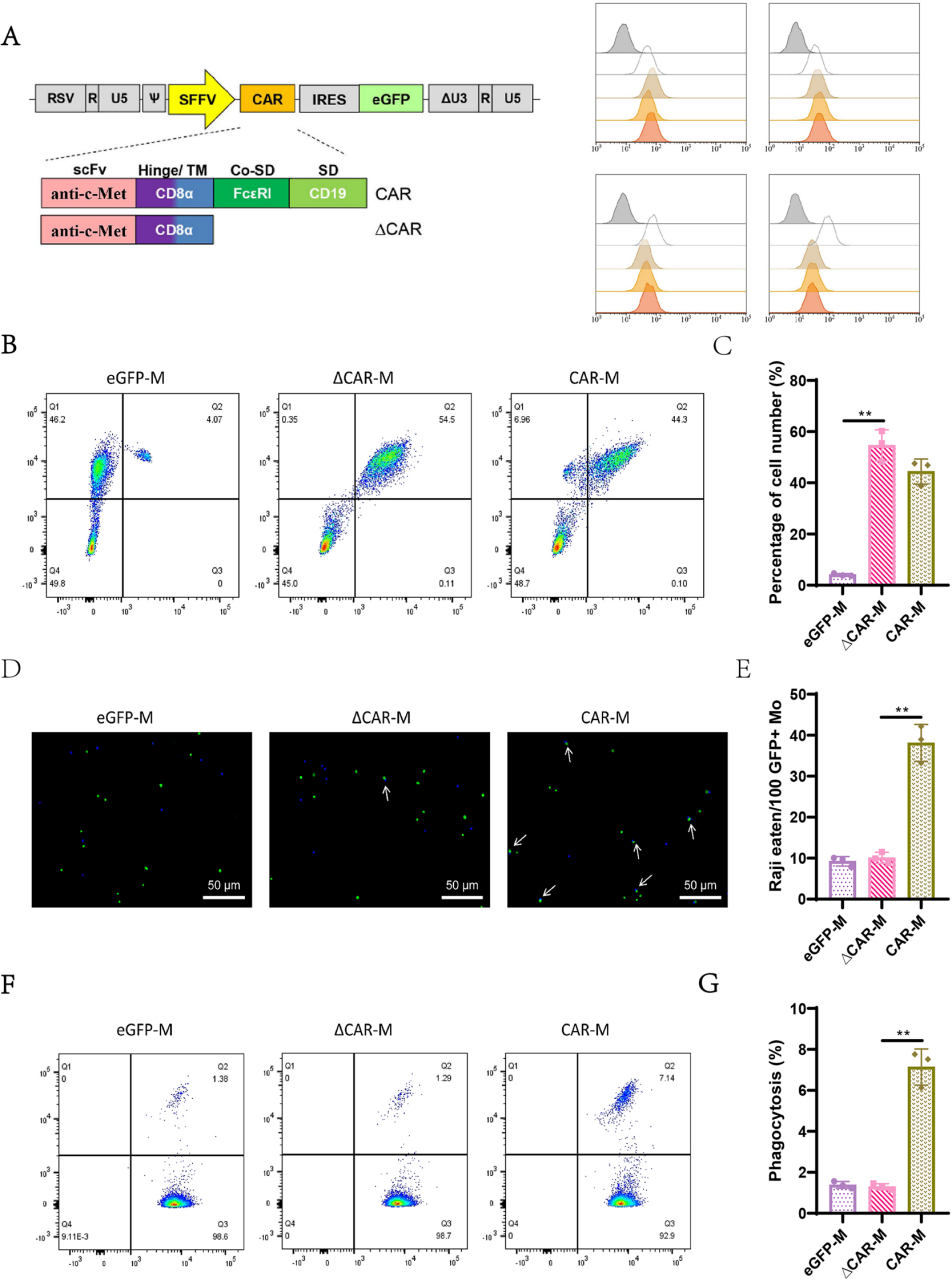
Construction and validation of c-Met specific CAR macrophages. *Note*: (**A**) Schematic representation of the anti-c-Met CAR (CAR-) and the truncated CAR (ΔCAR-) lacking the intracellular signaling domain. (**B**) Flow cytometry analysis detecting the Expression of GFP and CAR in eGFP-, ΔCAR-, and CAR- macrophages, with statistical data for each. (**C**) Flow cytometry analysis of surface markers on the three groups of macrophages. (**D**) Immunofluorescence staining observing the Phagocytosis of Raji cells by the three groups of macrophages, scale bar = 50 μm, with green fluorescence marking macrophages and red fluorescence marking Raji cells. A representative photo from one of three repeated experiments is shown. (**E**) Bar graph statistical analysis of phagocytosis action from (**D**), displaying the number of Raji cells phagocytosed per 100 GFP + macrophages. (**F**) Flow cytometry analysis of the phagophagocytosisaji cells by the three groups of macrophages, shoishowspical flow cytometry result from one of three repeated experiments. (**G**) Bar graph statistical analysis of the phagocytosis efficiency from (**F**), displaying the percentage of GFP + macrophages that phagocytosed Raji cells. *Note*: eGFP empty vector control (eGFP-M), macrophages with truncated CAR (ΔCAR-M), and macrophages with complete CAR (CAR-M). Quantitative data are presented as mean ± SEM, with p-values determined by two-tailed Student’s t-test, **, *p* < 0.01

To generate CAR macrophages from iPSCs, we employed lentiviral transduction using a second-generation CAR construct targeting c-Met. This CAR construct features a CD8a hinge and transmembrane domain alongside a tandem signaling domain comprising the common γ-chain of the Fc receptor and a c-Met recruiting domain (Fig. 3A). Expression of the CAR is driven by the SFFV promoter, linked to an eGFP reporter gene. For controls, we utilized a truncated form of the anti-c-Met CAR (∆CAR) lacking intracellular signaling domains and a vector expressing eGFP alone. Following differentiation, the iPSCs yielded three types of macrophages: eGFP-M, ∆CAR-M, and CAR-M. Flow cytometry analysis of these vectors based on eGFP and CD19 Expression indicated a transduction efficiency of approximately 50% (Fig. 3B). Further analysis of macrophage surface markers (CD45+, CD11b+, CD14+, and CD163+) confirmed the differentiation of iPSCs into mature macrophages across all three vector treatments (Fig. 3C), demonstrating that CAR expression does not impede macrophage differentiation or maturation.

To assess the anti-cancer efficacy of CAR-mediated targeting of c-Met + tumor cells, CAR-macrophages and control cells (eGFP-M, ∆CAR-M) were co-cultured with c-Met + mCherry-labeled Raji cells at a 1:1 ratio. After 4 h, fluorescence microscopy and image-based flow cytometry revealed co-localization phenotypes between Raji cells and c-Met CAR macrophages, indicating successful Phagocytosis (Fig. 3D and F). The phagocytic capability was quantified via flow cytometry, analyzing the percentage of mCherry + macrophages. Compared to the eGFP-M and ∆CAR-M groups, the CAR-M group exhibited significantly higher phagocytosis levels (Fig. 3E and G).

These findings demonstrate that our CAR design possesses high specificity and targeting capability against c-Met-expressing cells, laying a foundation for future functional experiments and clinical applications.

### CAR macrophages inhibit angiogenesis in pancreatic CSCs

**Fig. 4:**
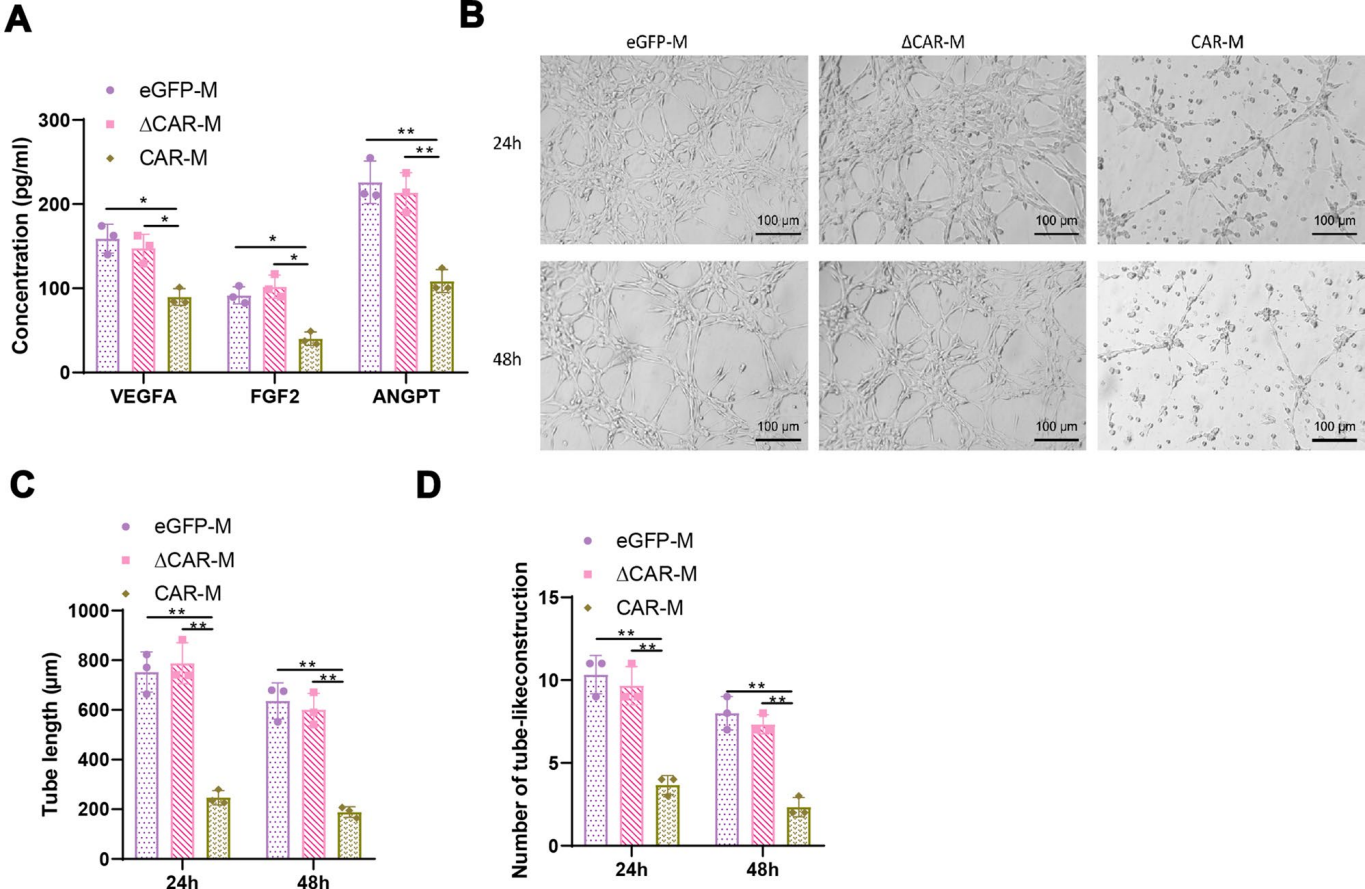
Potential of CAR macrophages to inhibit angiogenesis in vitro. *Note*: (**A**) Enzyme-linked immunosorbent assay (ELISA) measurement of angiogenesis-related cytokine levels (pg/ml). (**B**) Matrigel tube formation assay using culture medium supernatant from the three groups of macrophages and Raji cell co-culture treated HUVECs, with F-actin marked in green fluorescence. A representative photo from one of three repeated experiments is shown. (**C**) Quantitative analysis and bar graph of the total length of capillary-like structures formed in the Matrigel tube formation assay. (**D**) Statistical analysis and bar graph of the number of tube-like structures formed in the Matrigel tube formation assay. *Note*: Quantitative data are presented as mean ± SEM, with p-values determined by two-tailed Student’s t-test, **, *p* < 0.01

To elucidate the anticancer effects of CAR macrophages, we utilized ELISA to measure the expression levels of angiogenesis-related cytokines in the co-culture supernatant. The results showed significant reductions in VEGFA, FGF2, and ANGPT concentrations in the CAR-M group (Fig. 4A), suggesting that CAR macrophages might modulate the tumor microenvironment by regulating angiogenic factors. This hypothesis was further validated by an in vitro Matrigel tube formation assay. Compared to the eGFP-M and ∆CAR-M groups, HUVECs treated with supernatant from the CAR-M group exhibited markedly weakened tube formation capabilities, evidenced by significantly shorter total tube lengths (Fig. 4B-C) and reduced numbers of tubular structures (Fig. 4D), indicating that c-Met targeting CAR macrophages can suppress Angiogenesis. These findings imply that c-Met CAR macrophages may hinder tumor angiogenesis by inhibiting the release of angiogenesis-related cytokines.

### In vivo suppression of pancreatic cancer progression by CAR macrophages

**Fig. 5:**
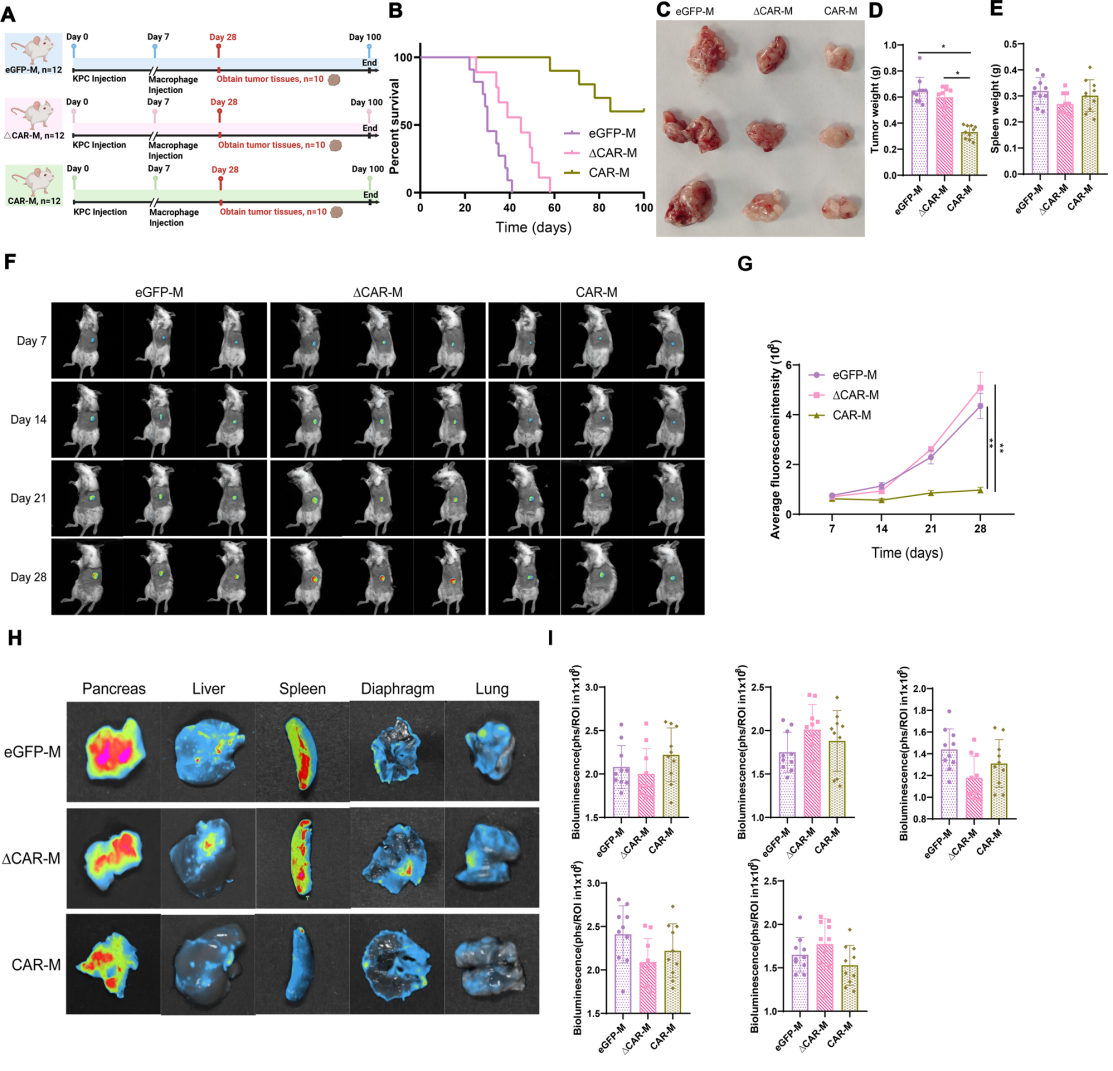
Inhibitory effect of CAR macrophages on pancreatic cancer growth in a mouse model. *Note*: (**A**) Treatment flowchart for NSG mice: Day 0, KPC cell injection to establish tumors; Day 7, injection of respective groups of macrophages; Day 28, tumor tissue collection from 10 mice per group; continued survival tracking for 100 days. (**B**) Tracking and Kaplan-Meier survival curve analysis for the three groups of mice. (**C**-**E**) Related measurements on Day 28 post-tumor tissue collection: (**C**) Tumor size measurement and photography, showing a representative photo from one of three repeated experiments; (**D**) Weight measurement of the harvested tumor tissue; (**E**) Spleen tissue weight measurement. (**F**) Bioluminescence imaging (BLI) is performed every 7 days on the three groups of mice, with all images displayed and a total of four measurements taken. (**G**) Quantitative analysis and curve plot of fluorescence intensity from Fig. 5F. (**H**) BLI of pancreas, liver, spleen, diaphragm, and lung from the three groups of mice on Day 28, with a circle around each organ indicating the same defined area for each image. A representative photo from one of three repeated experiments is shown. (**I**) Quantitative analysis and bar graph of fluorescence intensity from Fig. 5H. *Note*: Quantitative data are presented as mean ± SEM, with p-values determined by two-tailed Student’s t-test, *, *p* < 0.05; **, *p* < 0.01

To further investigate the anticancer effects of CAR-M in vivo, we conducted cell injections in mice with established tumors. Using a humane standard of care, female NSG mice aged 6 to 8 weeks were utilized to model pancreatic cancer, with successful tumor establishment using KPC cells expressing luciferase. After 7 days of model establishment, mice were randomly divided into three groups (each with 10 mice) and were subjected to intravenous injections of three types of macrophages (eGFP-M, ∆CAR-M, and CAR-M) (Fig. 5A). Their survival was monitored and survival curves were plotted. The experimental results demonstrated a significant extension of lifespan in the group injected with CAR-M compared to the eGFP-M and ∆CAR-M groups (Fig. 5B). Furthermore, based on the survival curves, mice were dissected at day 28, and tumor tissues were collected and measured, revealing a significant reduction in tumor volume in the CAR-M group compared to the other two groups (Fig. 5C). Further weighing demonstrated a significant reduction in tumor weight in the CAR-M group (Fig. 5D), with no significant changes in spleen weight across the three groups (Fig. 5E).

Using bioluminescence imaging (BLI), non-invasive monitoring of orthotopic tumor growth and metastasis was conducted in the remaining 3 mice from each group (Fig. 5A), with organ harvesting at the experiment’s end to confirm distant metastases. Since bioluminescence intensity measured in photons/second/region of interest (ph/s/ROI) is proportional to tumor load, BLI allowed for the quantitative assessment of tumor progression. Bi-weekly measurements revealed that post-tumor implantation, pancreatic tumor burden in CAR-M mice was significantly inhibited compared to eGFP-M and ∆CAR-M groups (Fig. 5F and G), indicating the in vivo tumor-suppressive effect of CAR-M. Additionally, on day 28, organs, including the pancreas, liver, spleen, diaphragm, and lungs, were collected for further BLI assessment of pancreatic cancer metastasis. The results showed significantly reduced bioluminescence intensity in organs from CAR-M mice, demonstrating CAR-M’s inhibitory effect on tumor metastasis in vivo (Fig. 5H and I).

### CAR macrophages inhibit tumor angiogenesis in mouse models of pancreatic cancer

**Fig. 6:**
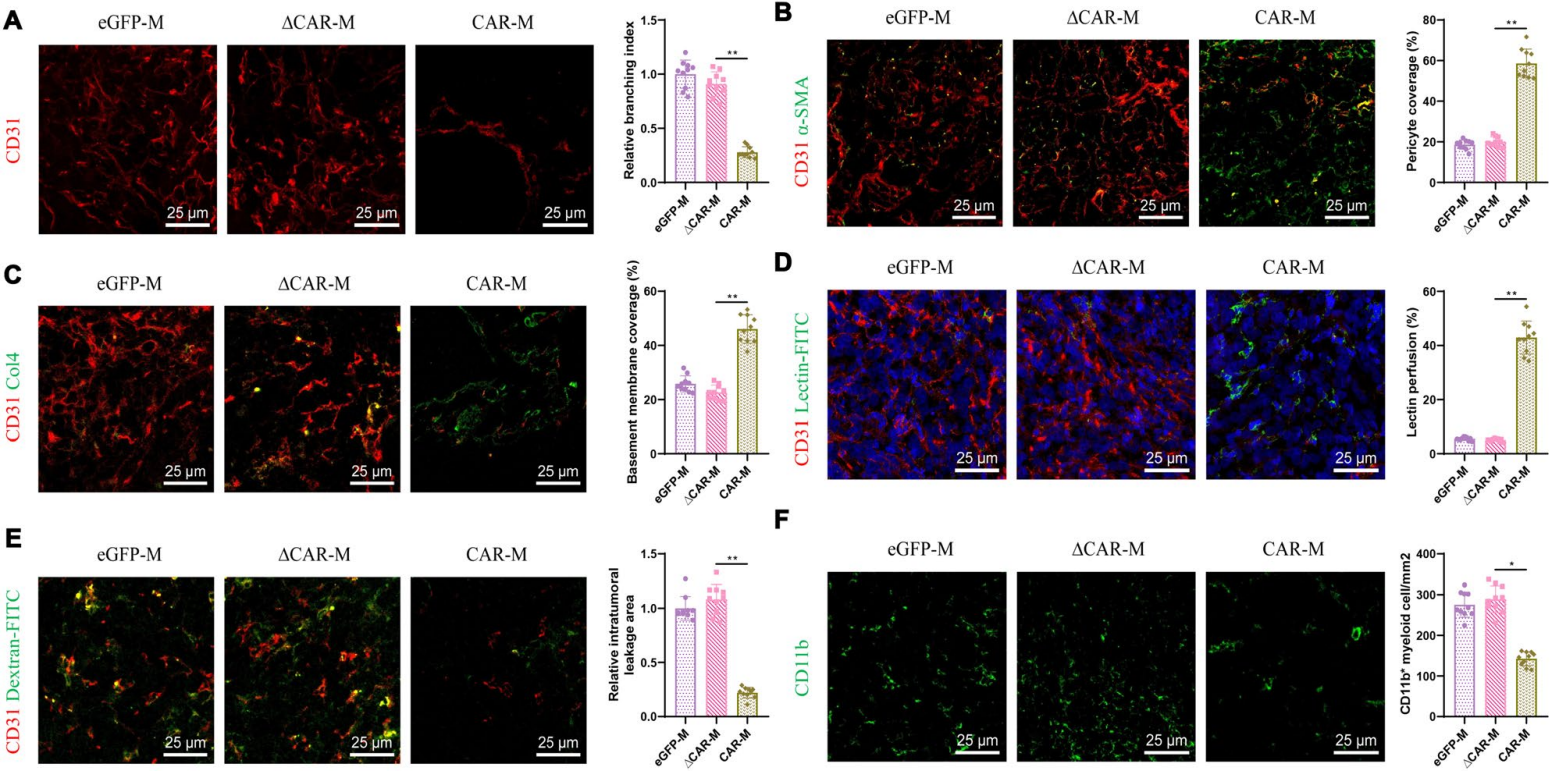
Impact of CAR macrophage treatment on tumor angiogenesis in pancreatic cancer mice. *Note*: (**A**–**C**) Immunofluorescence staining of CD31 (**A**), αSMA (**B**), and Collagen IV (**C**) in tumor sections from the three groups of mice, with quantitative statistical analysis and bar graphs for branching index (**A**), pericyte coverage (**B**), and basement membrane coverage (**C**) using ImageJ software. (**D**–**E**) Fluorescence microscopy observation of vascular lectin (**D**) and dextran (**E**) perfusion in tumor vessels post-treatment, with quantitative statistical analysis and bar graphs of the images using ImageJ software. (**F**) Immunofluorescence staining and photography of CD11b + myeloid cells in tumor sections, with statistical analysis and bar graph of CD11b + cell counts using ImageJ software. *Note*: Representative photos from one of six repeated experiments are shown. Quantitative data are presented as mean ± SEM, with *p*-values determined by two-tailed Student’s t-test, scale bar = 25 μm

To further elucidate the mechanism through which CAR macrophages inhibit tumor growth, we conducted immunofluorescence assays to examine tumor microvasculature in three groups of mice: eGFP-M, ∆CAR-M, and CAR-M. The results revealed a significant reduction in CD31-marked microvessels within the pancreatic cancer sites of mice treated with CAR-M (Fig. 6A), indicating a decrease in vascular density and angiogenic capability following treatment with c-Met CAR macrophages.

Moreover, compared to the control groups (eGFP-M and ∆CAR-M), mice injected with CAR-M displayed a significant increase in the fluorescence intensity of α-smooth muscle actin (α-SMA), a marker for pericytes, suggesting enhanced pericyte coverage and vascular smooth muscle activity (Fig. 6B). Additionally, the level of collagen IV (Col4), a component of the endothelial cell basement membrane (BM), was significantly higher in the tumor vessels of the CAR-M group (Fig. 6C). These indicators suggest that excessive Angiogenesis in the tumors was suppressed following treatment with c-Met CAR macrophages.

Furthermore, compared to controls, lectin perfusion experiments showed significant aggregation of lectin within the vasculature of the CAR-M group (Fig. 6D), and dextran perfusion experiments demonstrated a significant reduction in the area of dextran leakage within the tumor tissues of the CAR-M group (Fig. 6E). These results indicate a marked decrease in the high permeability characteristic of abnormal vessels, further proving that CAR-M treatment normalizes the vascular environment within mouse tumors.

Lastly, the infiltration of CD11b positive myeloid cells in tumors of the CAR-M group was significantly lower compared to controls (Fig. 6F), suggesting a reduction in tumor-associated inflammation and a more normalized tumor microenvironment. Therefore, we propose that treatment with c-Met CAR macrophages can alter the tumor microenvironment in vivo, inhibiting excessive and abnormal growth of tumor vessels.

Collectively, these findings demonstrate that c-Met CAR macrophages effectively inhibit Angiogenesis within the pancreatic cancer tumor microenvironment, promote normalization of tumor vasculature, and thereby effectively delay tumor progression.

## Discussion

This study provides an in-depth investigation into novel therapeutic strategies for pancreatic cancer, particularly focusing on the application of CAR macrophages targeting c-Met, utilizing single-cell multi-omics technologies. Unlike traditional research that emphasizes chemotherapy, radiotherapy, and targeted drug therapies, this study introduces a cell-based therapeutic approach, offering a novel perspective for pancreatic cancer treatment and shedding light on its complex biological traits. Moreover, it underscores the significance of single-cell multi-omics in unveiling tumor heterogeneity and microenvironmental interactions, thus laying a foundation for future precision medicine endeavors.

The application of CAR macrophage (CAR-M) therapy in pancreatic cancer treatment represents a significant advancement in immunotherapy (Zhang et al. 2023). Unlike traditional CAR-T cell therapy, CAR-M therapy has distinct advantages in solid tumors. Firstly, macrophages can naturally infiltrate the core regions of solid tumors, whereas CAR-T cells often face barriers posed by the tumor microenvironment (Maalej et al. 2023). Secondly, macrophages can perform antigen presentation and phagocytosis within the tumor, further activating adaptive immune responses locally and enhancing the overall antitumor immune effect (Klichinsky et al. 2020). Additionally, CAR-M therapy has shown unique efficacy in inhibiting tumor angiogenesis, especially through modulating the tumor microenvironment to block angiogenesis, potentially offering an advantage over traditional CAR-T cell therapies (Pan et al. 2022; Maalej et al. 2023; Chen et al. 2021). In this study, CAR macrophages specifically recognize and phagocytize c-Met-overexpressing pancreatic cancer cells, providing a new strategy to combat angiogenic mimicry in pancreatic cancer. Compared to existing immunotherapies, this approach theoretically offers greater target specificity and therapeutic potential.

Leveraging single-cell multi-omics, this research delves into the molecular mechanisms and cellular heterogeneity of pancreatic cancer, offering fresh insights for therapeutic strategy research. The detailed analysis at the single-cell level not only reveals intricate interactions within pancreatic cancer cells but also provides a rich repository of molecular targets for future investigations. This methodological innovation marks a departure from previous studies that relied on tissue or cell population-level analysis, highlighting the critical role of single-cell technologies in cancer research (Wu et al. 2021; Galeano Niño et al. 2022; Nalio Ramos et al. 2022).

The role of c-Met in pancreatic cancer and its potential as a therapeutic target constitute the core of this study. As a product of the proto-oncogene Met, c-Met plays a crucial role in cancer development. Previous research has shown that humanized c-Met antibodies can effectively inhibit invasion and induce apoptosis in NSCLC cells (Yamamoto et al. 1997). Klichinsky and colleagues pioneered the development of CAR macrophages for the treatment of solid tumors. They demonstrated the ability of CAR macrophages to specifically target and engulf tumor cells, as well as activate adaptive immunity, within humanized mouse models of the immune system (Höld et al. 1999).

In this study, we used the FindAllMarkers function to identify differentially expressed signature genes in pancreatic cancer ductal cell populations. After intersecting these genes with previously identified transcriptomic differentially expressed genes and pancreatic cancer-related genes obtained from GeneCards, only the c-MET gene remained. Utilizing the Kaplan-Meier Plotter, our analysis of c-MET expression and patient prognosis demonstrated that patients with high c-MET expression had significantly shorter survival compared to those with low expression, reinforcing its role as an independent prognostic indicator. This finding not only supports the rationale for selecting c-MET as a therapeutic target but also emphasizes its potential value in pancreatic cancer treatment. Building on this foundation and considering the high Expression of c-Met in pancreatic cancer patients, we developed c-Met-specific CAR macrophages to explore their impact on pancreatic cancer. By thoroughly analyzing the biological functions of c-Met and its role in the progression of pancreatic cancer, this study not only validates the efficacy of CAR macrophage therapy targeting c-Met but also solidifies c-Met’s position as a therapeutic target. This finding resonates with previous reports on c-Met as a cancer therapy target, further emphasizing the importance of targeted immunotherapy strategies (Remon et al. 2023; Yoshida et al. 2022; Dong et al. 2022).

Through in vitro and in vivo experiments, this study confirms the phagocytic activity of CAR-c-Met macrophages against pancreatic cancer cells and their effect in inhibiting angiogenesis mimicry. The results demonstrate that engineered CAR-c-Met macrophages can effectively engulf target cells in vitro and significantly inhibit tumor growth in pancreatic cancer mouse models. Moreover, the reduction in angiogenic factor secretion by CAR-c-Met macrophages further impedes vascular formation within the tumor microenvironment. These findings not only showcase the potential therapeutic effects of CAR macrophage therapy but also provide theoretical and experimental justifications for its application in treating pancreatic cancer and potentially a broader spectrum of solid tumors. Furthermore, the discoveries of this study offer critical reference points for further exploration of CAR macrophage therapy in clinical settings.

This study explores a novel targeting strategy for treating pancreatic ductal adenocarcinoma and demonstrates the potential therapeutic value of c-Met-targeted CAR macrophages. Targeted therapies are diversifying treatment options in pancreatic ductal adenocarcinoma; however, numerous studies have also attempted to target other related signaling molecules, such as EGFR, MEK, and PI3K (Qian et al. 2020). While some EGFR inhibitors like erlotinib and nimotuzumab have shown efficacy, others have failed to yield significant results in clinical trials for pancreatic ductal adenocarcinoma, suggesting potential resistance mechanisms within pancreatic ductal adenocarcinoma to EGFR inhibitors (Moll et al. 2018; Jacobsen et al. 2015). Moreover, clinical observations indicate that pancreatic ductal adenocarcinoma may activate alternative signaling pathways to circumvent EGFR inhibition, further reducing the efficacy of therapies that target only EGFR (Qian et al. 2020). Consequently, combining EGFR inhibitors with other multi-targeted agents could be a more promising approach (Philip et al. 2010). For instance, combining EGFR inhibitors with inhibitors of key signaling molecules like MEK and C-RAF could not only more comprehensively suppress tumor proliferation signals but also decrease the adaptive resistance mechanisms of tumor cells to single agents (Blasco et al. 2019; Smalley and Smalley 2018). These combination strategies show potential in preventing tumor escape and reducing resistance risk, warranting further research into optimized combinations and clinical efficacy.

In contrast, the c-Met-targeting strategy explored here, using CAR macrophages that directly target pancreatic ductal adenocarcinoma cells with high c-Met expression, exhibits high specificity and substantial tumor-suppressive effects. By blocking tumor angiogenesis and promoting phagocytosis of tumor cells, this strategy offers a new direction to address specificity and resistance issues in pancreatic ductal adenocarcinoma treatment and provides a scientific basis for future targeted therapy combinations.

Despite achieving notable results, the study faces limitations such as the complexity of efficacy assessments and uncertainties regarding long-term effects and safety. Future research is warranted in larger-scale clinical trials to validate the efficacy and safety of CAR-c-Met macrophage therapy and explore its potential in other types of solid tumors. Additionally, further optimization of CAR macrophage design and functionality to enhance their viability and therapeutic effect in complex tumor microenvironments remains a vital direction for future investigations.

## Conclusion

**Fig. 7:**
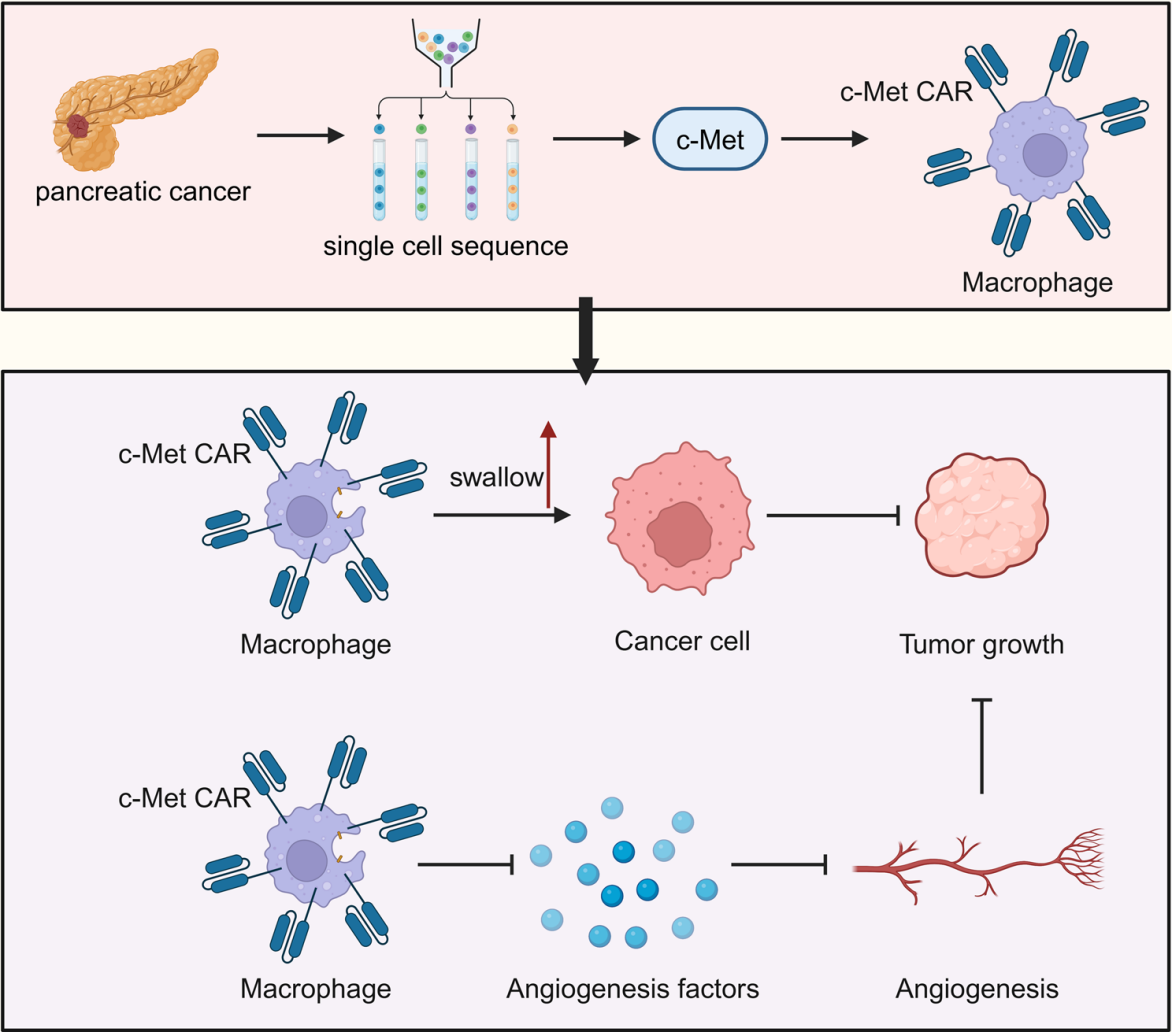
The role of CAR macrophages derived from pluripotent stem cells in pancreatic cancer development. In the immunological microenvironment of pancreatic cancer, an increase in immune cells, especially CAR macrophages, within the pancreatic cancer tissue (left image). Key genes like c-Met are upregulated in pancreatic cancer cells. Our designed CAR macrophages targeting c-Met effectively phagocytose pancreatic CSCs, inhibiting Angiogenesis and thereby suppressing tumor growth (right image)

By integrating single-cell multi-omics technology and the innovative application of CAR macrophage therapy, this study offers new strategies and insights for the treatment of pancreatic cancer. The CAR macrophages targeting c-Met exhibit potential therapeutic effects in inhibiting angiogenesis mimicry in pancreatic cancer, providing new directions for the research and treatment of pancreatic cancer and other solid tumors. Despite facing challenges and limitations, the findings of this study lay the groundwork for future cancer treatment research and clinical applications, holding significant scientific and clinical value (Fig. 7).

## Data Availability

Data are available from the corresponding author upon reasonable request.
